# Effect of educational interventions on health in childhood: A meta-analysis of randomized controlled trials: Retraction

**DOI:** 10.1097/MD.0000000000027006

**Published:** 2021-08-20

The article “Effect of educational interventions on health in childhood: A meta-analysis of randomized controlled trials”,^[1]^ which appeared in Volume 97, Issue 36 of *Medicine* is being retracted due to duplicate submission, duplicate publication, alleged falsification of authorship and alleged falsification of the License to Publish agreement.

*Medicine* became aware of the duplicate publication in Volume 164 of *Public Health*^[2]^ via reader concerns. Through a joint investigation, it was determined that authors submitted and published the same article in *Medicine* while it was under consideration for publication in *Public Health.* During the course of the investigation, several authors stated they were unaware of the publication. The authors alleged that their contributions and email addresses, including the corresponding author, were falsified by another author during submission. The License to Publish agreement, which requires the acknowledgement of the corresponding author, was also allegedly falsified.
